# Comparative Evaluation of Bacterial Reduction by Laser-Activated Irrigation Technique (LAI) With Conventional Needle Irrigation (CNI) in Single-Rooted Teeth With Pulpal Necrosis: A Single-Blinded Randomized Controlled Trial

**DOI:** 10.7759/cureus.50666

**Published:** 2023-12-17

**Authors:** Keerthika Rajamanickam, Sandhya Raghu, J Vijayashree Priyadharsini, Delphine Priscilla Antony, Nivedhitha Malli Sureshbabu

**Affiliations:** 1 Department of Conservative Dentistry and Endodontics, Saveetha Dental College and Hospitals, Saveetha Institute of Medical and Technical Sciences, Saveetha University, Chennai, IND; 2 Centre for Cellular and Molecular Research, Saveetha Dental College and Hospitals, Saveetha Institute of Medical and Technical Sciences, Saveetha University, Chennai, IND

**Keywords:** root canal disinfection, bacterial reduction, qpcr, novel technique, laser activated irrigation, conventional needle irrigation

## Abstract

Aim

To compare the bacterial reduction in single-rooted teeth with pulpal necrosis after laser-activated irrigation technique (LAI) and conventional needle irrigation (CNI).

Methodology

In this clinical trial (CTRI/2021/09/047767), 32 patients with pulpal necrosis were enrolled. Under complete aseptic conditions, access cavity preparation was done and the baseline sample S1 was collected from the root canal using paper points. After chemo-mechanical preparation they were allocated into two groups, following block randomization; Group A - CNI with 27 gauge side-vented needle, Group B - LAI with pulsed Er,Cr:YSGG (erbium, chromium:yttrium-scandium-gallium-garnet) (2780 nm) laser. After irrigant activation, canals were dried and a second sample S2 was taken using paper points. Microbial analysis using quantitative polymerase chain reaction (qPCR) was done to quantify the bacterial reduction among the two groups. Inter-group and intra-group analysis was done using the independent student t-test and Bonferroni test, respectively. The data was represented in terms of quantification cycle (Cq) values, which are inversely proportional to the microbial count.

Results

There was no significant difference in S1 between the two groups (mean difference=0.0205; p=0.912). There was a significant difference in S2 between the two groups for the organisms (mean difference=0.8042; p=0.000). The mean percentage of bacterial reduction in CNI was 10.82% and in LAI it was 25.92%.

There was a significant difference in S1 through S2 within the two groups for the organisms (p=0.000). The mean difference of Cq value is high for LAI compared to CNI (1.3494).

The fold change was calculated by taking the ΔCq value and ΔΔCq value after the logarithmic transformation of the Cq value. LAI showed lower levels of DNA at S2 similar to CNI. There is no significant difference in mean fold change between CNI and LAI (p=0.564).

Conclusion

This clinical trial concluded that both LAI and CNI were effective in bacterial reduction. There was greater bacterial reduction with LAI (25.92%) than with the CNI (10.82%) in single-rooted teeth with pulpal necrosis using qPCR analysis.

## Introduction

Biofilm, a complex structure with extracellular polymeric substances as a protective gel matrix is highly resistant to various antimicrobial agents used for root canal disinfection. The association between the persistence of biofilm and its sequel to apical periodontitis is well-known. Achieving complete debridement and instrumentation of all the surfaces of the root canal is practically impossible which could be attributed to anatomical variation such as the presence of complex root canal anatomy, isthmus, apical delta, lateral canals, etc. [1]. Thus to reach these inaccessible areas various irrigants and irrigant activation systems have been used [2]. Activation of irrigants has been suggested as the most effective solution for the problem that has led to the evolution from manual dynamic activation to laser activation systems [3].

The depth of bacterial penetration into the dentinal tubules has been found to be as far as 1250 mm [4]. Reasons why biofilm remains in situ can be attributed to conventional and mechanical instrumentation where 35-53% of the root canal surface remains untouched [5]. Chemical disinfection and mechanical debridement are commonly used modes to remove the organisms from root canals.

Sodium hypochlorite (NaOCl) at varying concentrations has been used as a root canal irrigant for its antimicrobial activity. Increasing the exposure time and temperature of NaOCl has been shown to improve its effectiveness. This allows its clinical application even at lower concentrations. Root canal disinfection with various irrigants is delivered with the conventional needle irrigation technique (CNI), which is not only passive but also has an inability to reach narrow anatomical features [6,7].

Laser is a promising tool with the ability to provide root canal disinfection. The literature review establishes the role of the multi-sonic ultra-cleaning system, erbium:yttrium-aluminum-garnet (Er:YAG) and erbium, chromium:yttrium-scandium-gallium-garnet (Er,Cr:YSGG) lasers, in tissue dissolution rate. The Er,Cr:YSGG laser has been researched in vitro and is identified to assist endodontic treatment without affecting periodontal tissues. It works on the principle of high absorption coefficients in hydroxyapatite (OH-) groups and water molecules, and consequent biophysical interactions [8-10].

In an aqueous solution, the shock waves were produced by the erbium lasers with radial firing tips. This induces primary and secondary cavitation effects in the aqueous solution which in turn supports debris and smear layer removal. The laser also enhances antimicrobial activity at a depth into dentin where root canal irrigants cannot penetrate [11].

To our knowledge, there are no published randomized clinical trials comparing Er,Cr:YSGG irradiation and conventional irrigation using NaOCl and Ethylenediaminetetraacetic acid (EDTA). Thus the aim of our study was to compare the bacterial reduction in teeth with pulpal necrosis after laser-activated irrigation technique (LAI) and CNI during root canal treatment. The objective of the study was to assess the quantitative reduction of bacteria in teeth with pulpal necrosis by means of quantitative polymerase chain reaction (qPCR) analysis after LAI and CNI and to evaluate bacterial reduction among the samples of both the groups; before the root canal preparation, and after the root canal preparation and irrigation protocols using qPCR analysis. The null hypothesis is that there is no difference in bacterial reduction in teeth with pulpal necrosis after being subjected to LAI and CNI during root canal treatment.

## Materials and methods

Study design

It was a single-blinded (evaluator), institutional-based (IHEC Ref No: IHEC/SDC/ENDO-1906/20/396) (SRB/SDC/ENDO-1906/20/TH-093), clinical trial registered under Clinical Trial Registry of India (CTRI/2021/09/047767). Block randomization and sequentially numbered, opaque, sealed envelope method of allocation concealment were carried out. The sample size was determined to be 32 using the G power 3.1 version (Heinrich Heine University, Düsseldorf, Germany) with a power of 99.9%. 

Eligibility Criteria

Inclusion criteria: Patients of 18-65 years with pulpal necrosis (Personal Activity Intelligence (PAI) Score: 1 - 2). Single-rooted teeth with single-canal.

Exclusion criteria: Intake of antibiotics within the previous three months. Intake of analgesics in the last 12 hours. Teeth with crown/ root fracture. Teeth with calcified pulp chamber or root resorption. Medically compromised individuals. Patients using any drugs for systemic illness. Aberrant anatomy.

Groups

Group A used CNI, while Group B used LAI.

Treatment procedure

Before the treatment, a careful medical and dental history was taken. Clinical examination included pulp sensibility tests, percussion and palpation testing, mobility, and pocket measurements. A radiographic examination was taken to rule out aberrant anatomy, resorption, calcifications, or periapical lesions. Only cases of pulpal necrosis (PAI score 1-2) were included in the study. Preoperative data for each patient was recorded in the predesigned patient’s chart which included tooth number, age, sex, and type of intervention used. The treatment and the study design were explained to the qualifying patients and informed consent was obtained from the voluntary patients who were willing to participate in the study. Patients who signed the informed consent were randomly assigned into two groups.

Conventional Root Canal Treatment

The single operator carried out all the clinical procedures. After confirming the pulpal status, teeth were isolated, and sterile carbide burs were used to prepare the access cavity under magnification. Composite build-up was done to serve as a chamber for irrigant delivery in case of proximal caries. An access cavity was prepared. To obtain the first sample (S1), a paper point was used to collect the root canal content. The baseline values (S1) thus were stored in Eppendorf tubes.

The basic endodontic procedure was carried out [12,13]. A glide path was established using stainless steel hand instruments. Working length determination was done using stainless steel hand K-files size #10 (Dentsply Maillefer, Switzerland) and the use of an apex locator (Root ZX II, J. Morita, Tokyo, Japan). It was confirmed using periapical radiographs and it was repeatedly checked throughout the procedure. All the canals were prepared using Protaper gold files (Dentsply Maillefer, Switzerland) to 0.5 mm short of the apex and size of #40 or #50 depending on the canal anatomy. The procedure was done following the manufacturer’s suggested sequence using a reduction gear handpiece powered by an electric motor (X-Smart, Dentsply Maillefer, Switzerland). Apical patency was maintained throughout the shaping procedure using #10 files (Dentsply Maillefer, Switzerland) between each instrument.

Irrigation Protocols

Irrigants used in all the groups were 5 ml of 3% NaOCl and 2 ml of 17% EDTA when shaping was completed.

Group A (CNI): Cleaning and shaping were done with conventional irrigation methods using a side-vented 27 gauge needle (Ultradent, South Jordan, United States). About 5 ml of 3% NaOCl was delivered into each canal, 1 mm short of the working length for 30 seconds with a flow rate of 0.1 mL s^−1^ approximately. After saline irrigation, 2 ml of 17% EDTA was delivered for one minute.

Group B (LAI): Laser safety protocol was followed. Cleaning and shaping were done with LAI (Waterlase iPlus Unit - Er:YSGG, 2780 nm - Waterlase iPlus Biolase Technolgy, San Clemente, United States). The laser beam was delivered using RFT tips (Endolase, Biolase Technolgy, Inc., Irvine, United States). About 5 ml of 3% NaOCl and 2 ml of 17% EDTA were used. Er:YSGG 2780 nm, RFT sizes 2 and 3, with settings 20 mJ per pulse, 50 microseconds pulse duration, and 400 W power were used. The laser tip was placed 2 mm short of working length and activated for 30 seconds for two cycles.

Paper points were used to dry the canals and the second sample S2 was taken. Calcium hydroxide intra-canal medicaments were carried to canals using a lentulo spiral (Dentsply Maillefer, Switzerland). The canal was sealed with a temporary coronal seal. In the following visit, obturation was done using AH plus sealer (Dentsply Maillefer, Switzerland) and 4% or 6% taper gutta-percha cones depending on the width of the canal. Lateral compaction was done in the case of wide canals. The tooth was then restored with a composite restoration.

Outcome measures

Absolute Quantification of Bacteria

PureLink genomic DNA Mini kit (Invitrogen, Carlsbad, United States) was used to isolate the Genomic DNA from the paper points collected. UV absorption at A260 (1.0 OD unit is equivalent to 50 μg) was used to quantify the DNA. The analytical grade 0.8% agarose gel was used for the quality analysis of genomic DNA. SYBR® Green-based method was used to quantify the bacterial load in the samples. The universal primers (16S ribosomal RNA), forward primer 5’-GATTAGATACCCTGGTAGTCCAC-3’ and reverse primer 5’-CCCGGGAACGTATTCACCG-3’ were used for in vitro amplification. The CFX96 Real-Time System (Bio-Rad Laboratories, Hercules, United States) was used for the purpose. A ten-fold dilution of standards was run in parallel for the calculation of the standard curve. The CFX Maestro software (Bio-Rad Laboratories, Hercules, United States) was used to analyze real-time polymerase chain reaction (PCR) data. The assessment was carried out by an assessor blinded to the samples received.

IBM SPSS Statistics for Windows, Version 23 (Released 2015; IBM Corp., Armonk, New York, United States) was used for statistical analysis. Inter-group and intra-group analysis was done using the independent student t-test and Bonferroni test, respectively, with a probability value of less than 0.05 as a significant level.

## Results

The data is represented in terms of quantification cycle (Cq) values, which are inversely proportional to the microbial count. 

Inter-group results

**Figure 1:**
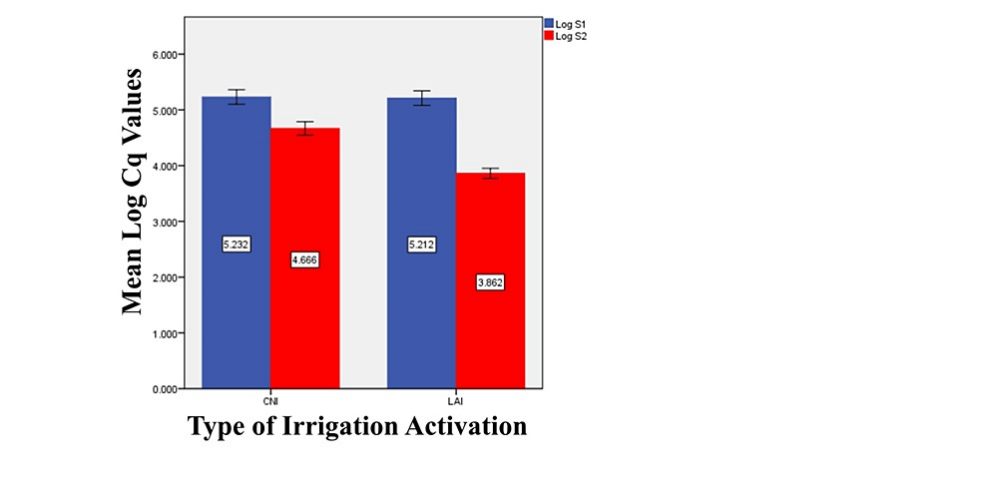
Mean comparison of total bacterial reduction between the groups Cq: Quantification cycle; S1: Baseline values; S2: After irrigation activation; CNI: Conventional needle irrigation; LAI: Laser-activated irrigation

Inter-group analysis using the independent student t-test showed a significant difference in S2 (after irrigant activation) between the two groups for the organisms (p=0.000) and there is no significant difference in S1 between the two groups (p=0.912). The mean Cq value is high for CNI compared to LAI in S2 with the mean percentage reduction of Cq in CNI is 10.82% and in LAI is 25.92% (Figure 1).

Intra-group results

**Figure 2:**
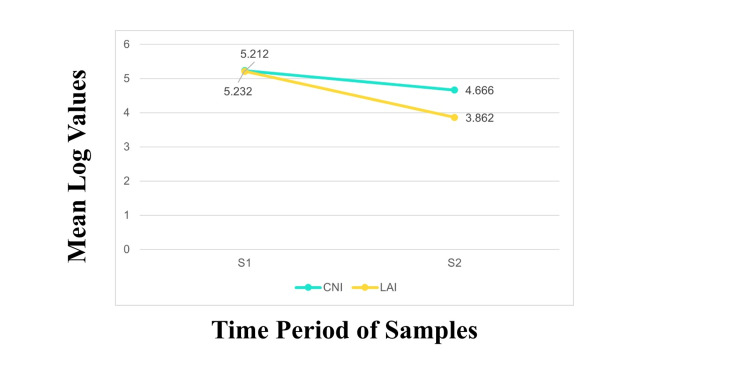
Mean comparison of total bacterial reduction within the groups S1: Baseline samples; S2: After irrigant activation; Cq: Quantification cycle; CNI: Conventional needle irrigation; LAI: Laser-activated irrigation

Intra-group comparison using the Bonferroni test showed a significant difference in S1 through S2 within the two groups for the organisms (p=0.000). The mean difference of Cq value was high for LAI (1.3500) compared to CNI (0.5660) (Figure 2).

Fold change

**Figure 3:**
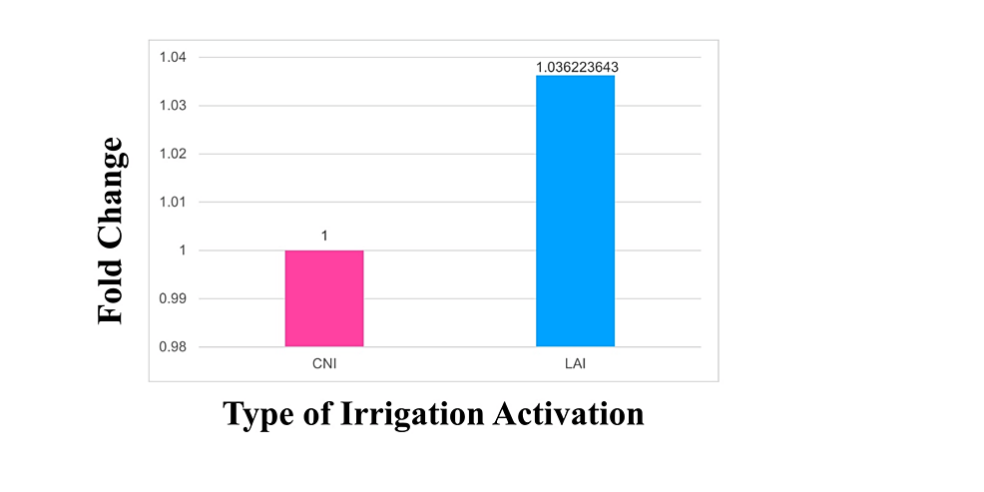
Mean fold change between the groups CNI: Conventional needle irrigation; LAI: Laser-activated irrigation

The fold change was calculated by taking the ΔCq value and ΔΔCq value after the logarithmic transformation of the Cq value. Then the 2^-ΔΔCq value was calculated. The average of this 2^-ΔΔCq value gives the fold change of S2 in the LAI group in comparison with CNI (control). LAI showed lower levels of DNA at S2 similar to CNI. There was no significant difference in mean fold change between CNI and LAI (p=0.564) thus proving that LAI can be used as efficiently as CNI (Figure 3).

## Discussion

Irrigation activation techniques promote the efficacy of irrigants even in mechanically inaccessible areas such as C-shaped canals and lateral canals. There is established evidence for microbial reduction, post-operative pain, debris removal, and clinical and radiological treatment outcomes by comparing irrigant activation techniques and CNI. There are clinical trials comparing various irrigant activation techniques such as sonic, passive, continuous ultrasonic, apical negative pressure irrigation, and manual dynamic agitation [14]. Positive pressure irrigation tends to push the irrigant in the periapical region and parameters such as gauge size, number, location and size of vents and the pressure of injecting irrigant, the position of the needle from apex while irrigation is factors to be considered for CNI [15,16].

On the assessment of the role of Er,Cr:YSGG on irrigant activation, it was identified that LAI was more efficient than PUI at boosting the antibacterial activity of 0.5% NaOCl against *Enterococcus faecalis* biofilm that had been present for 10 days. It had effectiveness comparable with 2.5% NaOCl [17].

The selection of an apical patency file minimizes the irritation to periapical tissues and minimizes post-operative discomfort [18]. Apex locator measurements were reconfirmed radiographically to limit the instrumentation within the root canal system [19]. Protaper gold files were chosen for instrumentation to reduce the chances of debris extrusion and the crown down technique was followed up to 1 mm short of working length [20].

To standardize the evaluation of lasers in clinical scenarios, single-rooted teeth were chosen for the study. The protocol of rubber dam isolation and pre-endo build-up was done in cases of proximal caries to ensure adequate disinfection and facilitate irrigant activation without discomfort to the patient [21] and occlusal reduction was done uniformly before recording the working length [22]. 

In CNI the delivery tip was positioned 1 mm short of working length as a safety measure [23]. Following the irrigation protocol, the patient was given an intermediate restoration with intracanal medication [24] and the obturation was done on the second visit [25].

LAI has a significant role in mechanically dislodging the endodontic biofilm, removal of the smear layer and optimally disinfecting the root canals. Using LAI, the amount of apical irrigant solution extruded was found to be significantly lesser than CNI [26]. The Er,Cr:YSGG laser produced the best results for thorough root canal disinfection when used in dry environments which could be attributed to less water content inside the canal leading to deeper penetration [27]. The temperature increases brought on by Er,Cr:YSGG laser irradiation at the settings used in this study have shown to be effective at removing debris and smear layers while preventing carbonization and melting [8].

Water absorbs the laser energy, producing vapor bubbles that expand and produce micro-cavitation effects. These effects were demonstrated to remove the smear layer and dislodge the microbial biofilm even in curved canals using dye removal techniques with radial firing tips [28].

Better debris removal was often achieved by placing the fiber tip closer to the apical region, increasing the irradiation period, shortening the pulse length, and raising the pulse energy. A higher pulse frequency had comparatively little effect [29]. Er:YSGG with settings 20 mJ per pulse, 50 microseconds pulse duration, and 400 W power were used in this study. The laser tip was placed 2 mm short of working length and activated for 30 seconds for two cycles. Er,Cr:YSGG laser with NaOCl solution demonstrated greater effectiveness in endodontics than when used alone. Significant reduction of microbial load at a depth of 200 μm into the dentinal tubules was achieved with the irrigant activation using Er,Cr:YSGG laser [30].

Er:YAG laser and passive ultrasonic irrigation were equally effective in reducing post-operative pain but the cases selected for the study were asymptomatic teeth [12]. This clinical trial observed that both LAI and CNI were effective in bacterial reduction. There was greater bacterial reduction with LAI (25.92%) than with the CNI (10.82%) in single-rooted teeth with pulpal necrosis using qPCR analysis. A previous study has also demonstrated that Er,Cr:YSGG laser treatment was as effective as 5% NaOCl in its bactericidal effect against *E. faecalis* [9]. Moreover, Er,Cr:YSGG has shown a faster tissue dissolution rate compared to conventional irrigation methods [10]. In necrotic teeth with chronic apical periodontitis, periapical healing after six months was reported to be similar with the use of Er,Cr:YSGG, or 3% NaOCl during root canal treatment [13]. The clinical application of Er,Cr:YSGG during root canal treatment can be recommended to overcome the adverse effects of chemical irrigants.

The microbial analysis was done using PCR analysis, which adds strength to the study because of its higher accuracy and reliability. The data is represented in terms of Cq values, which are inversely proportional to the microbial count. More the cycles required to quantify, the lesser the microbial load. The fold change was calculated by taking the Δcq value and ΔΔcq value after the logarithmic transformation of the Cq value. Then the 2^-ΔΔcq value was calculated. Thus indicating the large difference between the LAI and CNI in the microbial load.

Limitations of the clinical trial were that additional groups comparing sonic, ultrasonic, and other LAI were not included. The study did not include multi-rooted teeth or curved canals. For PCR analysis, species-specific primers required to identify the specific bacteria that might cause reinfection were not used. 

With the promising results of our clinical trial, we further recommend future studies using Er,Cr:YSGG laser for all endodontic treatment in both single and multi-rooted teeth with usage of various irrigants and evaluation using species-specific primers for more specific effect of lasers on the microorganism.

## Conclusions

This clinical trial concluded that both LAI and CNI were effective in bacterial reduction. There was greater bacterial reduction with LAI (25.92%) than with CNI (10.82%) in single-rooted teeth with pulpal necrosis based on qPCR analysis. Er,Cr:YSGG laser activation of irrigant has shown to be effective in root canal disinfection. 
